# Detecting Parkinson’s Disease through Gait Measures Using Machine Learning

**DOI:** 10.3390/diagnostics12102404

**Published:** 2022-10-03

**Authors:** Alex Li, Chenyu Li

**Affiliations:** 1 Stanford Center for Professional Development, Stanford University, Stanford, CA 94305, USA; 2 Department of Biomedical Data Science, Stanford University, Stanford, CA 94305, USA

**Keywords:** Parkinson’s disease, machine learning, gait measures

## Abstract

Parkinson’s disease (PD) is one of the most common long-term degenerative movement disorders that affects the motor system. This progressive nervous system disorder affects nearly one million Americans, and more than 20,000 new cases are diagnosed each year. PD is a chronic and progressive painful neurological disorder and usually people with PD live 10 to 20 years after being diagnosed. PD is diagnosed based on the identification of motor signs of bradykinesia, rigidity, tremor, and postural instability. Though several attempts have been made to develop explicit diagnostic criteria, this is still largely unrevealed. In this manuscript, we aim to build a classifier with gait data from Parkinson patients and healthy controls using machine learning methods. The classifier could help facilitate a more accurate and cost-effective diagnostic method. The input to our algorithm is the Gait in Parkinson’s Disease dataset published on PhysioNet containing force sensor data as the measurement of gait from 92 healthy subjects and 214 patients with idiopathic Parkinson’s Disease. Different machine learning methods, including logistic regression, SVM, decision tree, KNN were tested to output a predicted classification of Parkinson patients and healthy controls. Baseline models including frequency domain method can reach similar performance and may be another good approach for the PD diagnostics.

## 1. Introduction

Parkinson’s disease causes uncontrollable movements which are due to brain disorders. With the progression of this disease, patients can suffer from difficulty walking and talking, and mental and behavioral changes. The nerve cells in the patients’ basal ganglia who suffer from Parkinson’s disease become impaired and/or die, and this leads to less production of dopamine which is the key chemical messenger of the nervous system to control various functions of the body [1,2,3]. Four main symptoms exist in Parkinson’s patients, including tremor in arms, legs, heads and hands, muscle contraction for a long time, slowness of movement, impaired balance with falls sometimes. Among every 100,000 who are 80 or older, 1900 patients suffer from Parkinson’s disease. Though Parkinson’s disease is the most common among elder people, there are no specific tests to diagnose Parkinson’s disease [1,2,3]. The current state-of-the-art method is based on a patient’s medical history, a review of signs and symptoms, together with a neurological and physical check by a neurologist [4,5,6]. Though a dopamine transporter scan (DaTscan) can be a supportive test to identify integrity of the striatal dopaminergic system, the final diagnosis is still based on symptoms and neurologic examination [7,8,9,10]. Diagnostic accuracy is still not optimal with clinical features, diagnostic tests or biomarkers [11]. The early detection of PD is largely unmet while therapies can have a better chance for success in the early stage [12,13].

The gait dataset was used in one previous study by Hausdorff JM’s group [14] where they collected the data from sensors and described the gait speed characteristics in PD and controls using statistical methods. Another study from the same group [15] tested the comparison of gait speed on level ground and treadmill of PD and controls but mostly used descriptive analysis. Previous studies also used a statistical approach to test rhythmic auditory stimulation function on PD gait traces and concluded the potential usage of rhythmic auditory stimulation (RAS) as an intervention to improve mobility and reduce fall risk of PD patients [16,17]. Yogev G and colleagues [18] investigated the associations between executive function and gait variability and suggested a decline in executive function in PD. 

One recent publication [19] showed a successful approach using a random forest classification method to identify familial hypercholesterolemia with electronic health record data. This reveals the potentially important application of machine learning methods in disease diagnosis and high-risk patient identification. Some previous machine learning approaches focused on magnetic resonance imaging [20], motor features [21] and non-motor features [22]. There have been some machine learning trials tested on Parkinson’s disease. Machine learning methods are tested to PD datasets, including handwritten patterns [23], neuroimaging [24], cerebrospinal fluid [16], serum [25] and voice [26]. Jeon et al. [27] applied Principal Component Analysis (PCA) to the Spatial-Temporal Image of Plantar Pressure and used Support Vector Machine (SVM) to classify Parkinson gait and normal gait, but the sophisticated foot pressure system may be less accessible. Zhao et al. [28] and El Maachi et al. [29] proposed Convolutional Neural Network (CNN) approaches in classifying PD patients and healthy controls using the same dataset as us. While favorable results were achieved, the complexity of the deep neural networks is high and the associated training process can be expensive. Our study may reveal a possible usage of baseline machine learning classifiers for PD patient diagnosis that is cost effective, and provide insights on novel criteria for PD.

## 2. Materials and Methods

### 2.1. Dataset and Features

Gait in Parkinson’s Disease dataset [30,31] published by Jeffrey Hausdorff on PhysioNet is a database that contains measures of gait from 306 subjects. 214 of them are PD patients and 92 are healthy controls. The records include the vertical ground reaction force of subjects as they walked at their usual, self-selected pace for approximately 2 min on level ground. One’s gait is measured via 8 sensors underneath each foot that measure the vertical ground reaction force at a rate of 100 samples per second per sensor while the subject walks for a short period of time at their own pace. Therefore, each subject is in a multi-dimensional time series representation of k by 16 matrices where k is the number of force values collected in 0.01 s granularity for this particular subject. 

For baseline model, we used the full dataset. For frequency domain, decision tree, KNN methods, since the gait of each patient was measured for slightly different amount of time, we filtered the dataset by keeping the same time series length (8000 data points, or 80 s) and drop subjects who have fewer, resulting in a smaller dataset of 270 subjects with 186 being PD patients and 84 being healthy controls. In order to tune hyperparameters, the training set is further divided into training and validation sets using a 80:20 split with the same strategy. The study was repeated 10 times with different random selections of PD patients and healthy controls to obtain the mean and standard deviations of all metrics.

**Figure 1:**
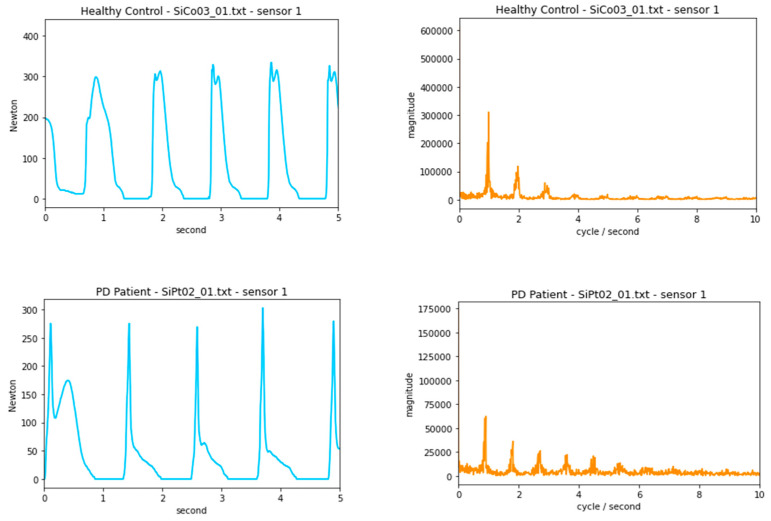
Time domain and frequency domain data from one sensor of a healthy control and a PD patient.

Figure 1 shows a subset of the time domain and frequency domain data from one sensor of a healthy control and a PD patient. With a spectral analysis, the peaks in the frequency domain indicate the dominant frequency of approximately once per second, which matches the time domain and is within our expectation of normal walking speed [32]. The plot of the PD patients shows greater uncertainty and noise in the frequency domain, likely indicative of motor system-related issues [33].

### 2.2. Baseline Model

Our baseline models draw inspiration from Yogev G., et al.’s prior work. Coefficient of Variation is a measure of variability as ratio of the standard deviation to the mean, and thus motor-system related symptoms are likely to be reflected on such metrics [34]. Since it measures the dispersion of the data with respect to its mean, it works well on the data series obtained from different patients. The force data are preprocessed to obtain the Coefficient of Variation $CV_{j}^{\left(i\right)}=\frac{\sigma_{j}^{\left(i\right)}}{\mu_{j}^{\left(i\right)}}$ for each sensor *j* of subject *i*, where σ is the standard deviation and μ is the mean of the data. Thus, each subject is represented by a one-dimensional vector of $x^{\left(i\right)}=CV^{\left(i\right)}\in R^{16}$. The CV metrics are then trained using Logistic Regression and Support Vector Machine with the Radial Basis Function (RBF) kernel, both with L2 regularization. The former minimizes the loss function where h(x)=g(θx)=1/(1+exp(−θx))  is the sigmoid function and θ are learned parameters. The regularization strength *λ* is a hyperparameter to be optimized.

$$\underset{i}{\sum }y^{\left(i\right)}\log h\left(x^{\left(i\right)}\right)+(1-y^{\left(i\right)}\log\left(1-h\left(x^{\left(i\right)}\right)\right)+\lambda \underset{j}{\sum }\theta_{j}^{2}$$

The latter solves the optimization problem for *w* and *b* in the hypothesis $g\left(w^{T}x+b\right)$ where *g* is the sign function. The regularization strength C and the hyperparameter γ of the RBF kernel are optimized using the validation set.

$$min_{w,b,\xi }\frac{1}{2}{\left|\left|w\right|\right|}^{2}+C\underset{i}{\sum }\xi_{i}^{2}\;s.t.\;y^{\left(i\right)}\left(w^{T}x^{\left(i\right)}+b\right)\geq 1-\xi_{i},\;\xi_{i}\geq 0,\;i=1,\ldots ,n$$

### 2.3. Frequency Domain

Walking can be seen as a periodic motion, so each patient’s data can be treated as a finite sequence of equally-space samples of some function [35]. Therefore, the vertical reaction force time series data can be approximated using the following model and turned into its frequency domain using discrete Fourier transform, where *N* = 8000 is the length of the time series and *i* is the imaginary unit. Since the force data are real values, we will use the modulus of only *k* = 0, 1, …, 3999. Therefore, the frequency domain representation of each patient has 16 × 4000 real non-negative values and will be evaluated using Logistic Regression and SVM.

$$X_{k}=\overset{N-1}{\underset{n=0}{\sum }}x_{n}\left(cos\frac{2\pi kn}{N}-isin\frac{2\pi kn}{N}\right),\;\;\;\;\;\;k=0,\;1,\;\ldots ,\;N-1$$

### 2.4. Decision Tree, K-Nearest Neighbors, Convolutional Neural Networks

Decision Tree is a non-parametric supervised learning method that learns the decision rules, which can be visualized in a tree-like structure for binary classification. Filtered dataset of 8000 datapoints for each of the 16 sensors was utilized in training this model. The input dataset for each subject is one-dimensional and 8000 × 16 in length. Sklearn DecisionTree Classifier function was applied and the related parameters were tuned to get the best model performance, including criterion, splitter, max depth, min samples split, random state and max features [36]. Decision trees tend to be unstable with small datasets [37], which was confirmed in our experiments.

K-Nearest Neighbors (KNN) is another non-parametric supervised learning method suitable for binary classification which relies on an assumption that similar data points are close enough [38]. In the KNN model, the same dataset with 8000 time points for each of the 16 sensors was utilized. The input dataset for each subject is one-dimensional and 8000 × 16 in length. SKlearn function KNeighborsClassifier was used and parameters were turned to get the best model performance (n neighbors, weights, algorithm, leaf size, metric and n jobs). Compared with the Decision Tree method, KNN reduces the overfitting issue and improves accuracy.

### 2.5. Limited Dataset

**Figure 2:**
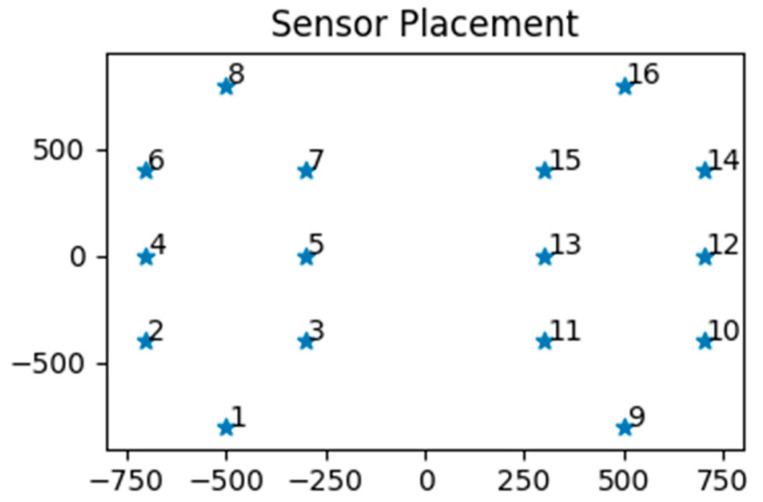
Relative placement of 16 under-foot sensors (scale is arbitrary).

As an extension, we also experimented with the case where there is only a subset of sensors available. If the models can still reliably predict Parkinson gaits versus regular gaits, it could help facilitate even simpler equipment in collecting vertical reaction force data and diagnosing PD. Specifically, the following two schemes were chosen (Figure 2).

- Sides-Only: all except sensors 1, 8, 9, 16
- Exclude-Diagonals: sensors 1, 4, 5, 8, 9, 12, 13, 16

The intuition behind these choices has to do with the possible correlation of the forces exerted by each region of the foot. These two subsets of the original data were to be evaluated using Logistic Regression and SVM.

## 3. Results

### 3.1. Baseline Model

With the healthy control as the negative samples and PD patients as the positive samples, we evaluate the predictions for this classification task with

- True Negative (TN): healthy control correctly identified
- False Positive (FP): healthy control incorrectly identified as PD
- True Positive (TP): PD patient correctly identified
- False Negative (FN): PD patient incorrectly identified as healthy control
- And the metrics
- Precision = TP/(TP + FP)
- Recall = TP/(TP + FN)
- False Positive Rate = FP/(FP + TN).

The SVM method on Coefficient of Variation achieved better training and test accuracy than the Logistic Regression (Table 1, Supplementary Table S2). Both models had close to zero false negative rate, but Logistic Regression had more trouble predicting over the negative examples compared to SVM, achieving about 50:50 between true negatives and false positives. Hyperparameters *λ* = 1 for Logistic Regression and C = 0.1, γ = 10 for SVM were found via grid search (Table 1, Supplementary Table S1). 

Since the dataset has an unbalanced positive and negative example, it is important to analyze beyond just accuracy. The near perfect recall score indicates good performance on the positive examples.

**Figure 3:**
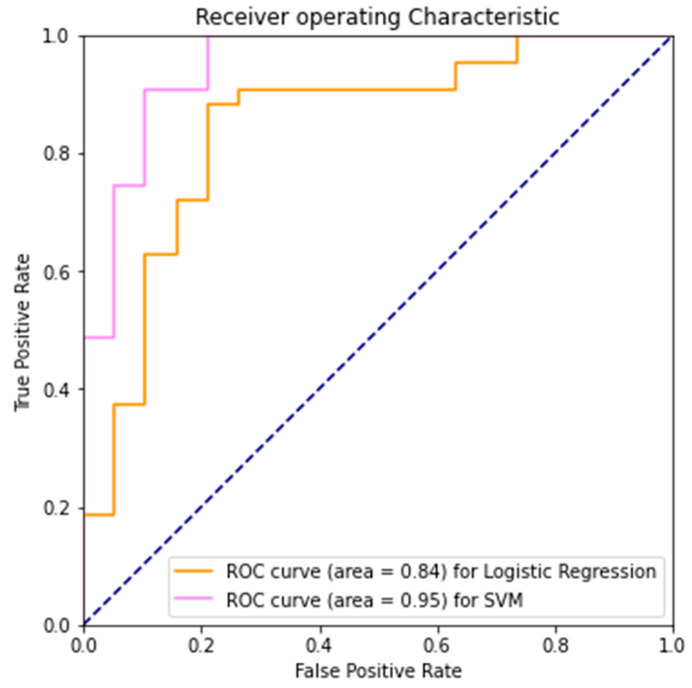
Presented Logistic Regression and SVM AUROC using the baseline model.

It is also valuable to look at its discriminatory ability, i.e., how well it can separate the positive and negative classes. The True Positive Rate (TPR) is the same as the Recall value, and the False Positive Rate (FPR) is higher than desired, which indicates less than optimal performance over the negative examples. By varying the decision thresholds, we can visualize the trade-off between TPR and FPR, the proportion of PD patients correctly classified and the proportion of healthy controls incorrectly classified, in the Receiver Operating Characteristic (ROC) curve. Area Under Receiver Operating Characteristic (AUROC) indicates the degree of separability; the higher the value, the more certain the model is in predicting each group. SVM’s higher AUROC indicates a superior performance in its ability to distinguish the two classes here (Figure 3).

### 3.2. Frequency Domain

The time series of 8000 data points (80 s) for each sensor of each patient was transformed into its frequency domain using discrete Fourier transform. Since the force data was sampled at once per 0.01 s granularity, the highest frequency detected is 1 cycle/0.02 s, or 50 cycles/s, which should give great insight into the subject’s gait. Since the time domain data is real, we only need the first half of the frequency domain since the rest are symmetrically redundant in their magnitude [39]. Therefore, we obtained 4000 frequency bins spaced at the reciprocal of time series length apart, 1/8000, for every sensor, ready to be trained. Hyperparamters *λ* = 1 for Logistic Regression and C = 0.1, γ = 1 × 10^−6^ for SVM were found via grid search (Table 1).

**Figure 4:**
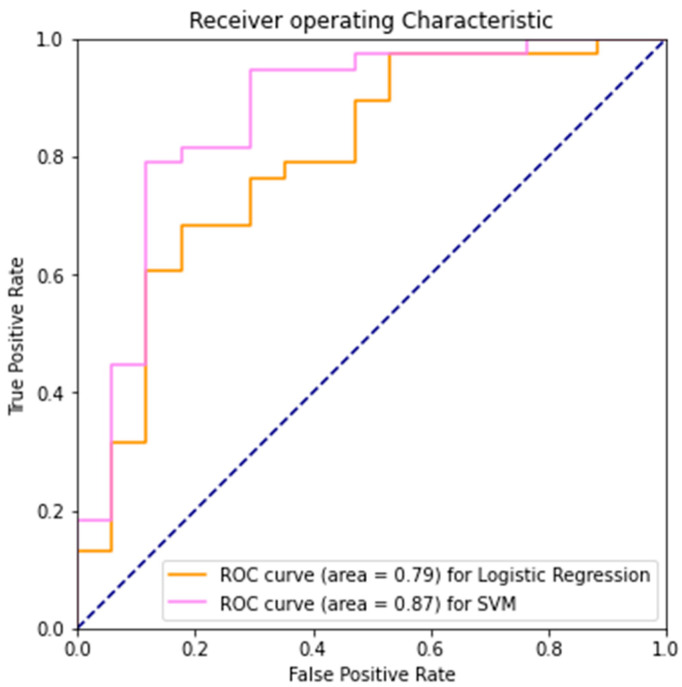
Presented Logistic Regression and SVM AUROC using Frequency Domain.

Logistic Regression achieved perfect accuracy on the training set and comparable test accuracy as the CV method in the baseline, whereas SVM with RBF Kernel per-formed better in test accuracy and recall. In terms of the ability to separate the two classes, AUROC still indicates SVM’s slightly better performance in this area (Table 1, Supplementary Table S2 and Figure 4).

### 3.3. Decision Tree

Decision tree builds qualitative classification models fitting the underlying distribution of data. The 8000 data points for each sensor are utilized to train the decision tree classifier. We turned parameters and the results indicated the best evaluation metric with a max depth of 43 (mean 43.1 with standard deviation 2.85), criterion as entropy and random splitter. It reached 0.60 mean accuracy with 0.71 mean precision and 0.70 mean recall score (Table 1, Supplementary Table S2). Decision tree did not improve the baseline methods performance, and this indicates better performance of logistic regression in small datasets.

### 3.4. K-Nearest Neighbors

The other non-parametric classification method used was KNN. Different numbers of neighbors from 1 to 10, different weights, and other parameters were evaluated, and 5 neighbors (mean 4.6 with standard deviation 0.70) with weights as distance provided the best performance with accuracy 0.70 with standard deviation of 0.05, precision 0.73 with standard deviation of 0.03 and recall 0.91 with standard deviation of 0.05 (Table 1, Supplementary Table S2). KNN performance is improved compared with decision tree, but does not exceed logistic regression. 

### 3.5. Limited Dataset

In both Sides-Only and Exclude-Diagonals schemes, Logistic Regression and SVM achieved slightly lower performance, which are expected due to some features being removed, but higher performance than decision tree and KNN. Logistic Regression and SVM both still maintained high recall (Table 2). 

## 4. Discussion

In our study, the baseline models with Coefficient of Variation using simple Logistic Regression and Support Vector Machine with the Radial Basis Function kernel already performed very well in the PD patient population, achieving a perfect recall score. The more complex models we used were not able to improve on the false positive rate and precision. Even though techniques such as regularization and early stopping have been applied to our more complex models, the training and test scores still suggest some overfitting due to the small dataset size. While the performance of our classifiers did not surpass that of the CNN models in the previous studies [28,29], our baseline models provide additional insights into PD diagnosis and the simplicity makes them more accessible.

All of our models have shown high values in the recall score, especially baseline models, indicating their excellent performance in identifying the PD group. This makes Parkinson’s disease detection through gait measures an effective first line of screening, and its non-invasive nature could expedite its acceptance in the field. Taken together previous studies in the field, this indicates that different machine learning methods can be good approaches to address PD diagnosis with clear criteria for patients. 

Moreover, the limited data exploration suggests that having fewer sensors would not significantly decrease the performance of the tested models. This could facilitate simpler and lower cost setups than the 16-sensor equipment used to obtain the dataset here and allow the technology to be more accessible. With the limited dataset, the overall performance is comparatively worse in the healthy subject population, suggesting some degree of variations in the gait profile of healthy adults as investigated in some studies [39,40].

Some limitations of this study include the small number of PD patients and healthy controls who came from similar demographics. In future work, possible directions in improving the performance of diagnosing PD via gait measures would include obtaining data from more healthy controls and PD patients so that deep learning methods can be better evaluated, as well as applying experimental deep network models that have improved capabilities with small datasets. Healthy gait measures can be further analyzed to understand their variations and help develop methods that can perform better without lowering the high recall scores in this paper. Also, external datasets with more even class distribution are required for evaluation to reduce bias in the current study. There may also be interesting properties in the frequency domain of the gait measure that can be learned by both non-deep and deep learning approaches. With further approaches, machine learning classifiers for PD patient diagnosis can be cost-effective with potential novel criteria.

## Figures and Tables

**Table 1:** Performance metrics of the test set.

		Accuracy Mean (SD) n = 10	Precision Mean (SD) n = 10	Recall Mean (SD) n = 10	False Positive Rate Mean (SD) n = 10
Baseline model	LR	0.81 (0.03)	0.81 (0.03)	0.95 (0.03)	0.52 (0.09)
	SVM	0.85 (0.03)	0.86 (0.03)	0.94 (0.04)	0.35 (0.09)
Frequency domain	LR	0.80 (0.04)	0.84 (0.03)	0.88 (0.05)	0.37 (0.09)
	SVM	0.84 (0.03)	0.84 (0.03)	0.95 (0.05)	0.41 (0.10)
	DT	0.60 (0.06)	0.71 (0.04)	0.70 (0.08)	0.64 (0.12)
	KNN	0.70 (0.05)	0.73 (0.03)	0.91 (0.05)	0.75 (0.10)

Tuned hyperparameters for baseline model LR: λ = 1; baseline model SVM: C = 0.1, γ = 10; frequency domain LR: λ = 1; frequency domain SVM: C = 0.1, γ = 1 × 10^−6^; DT: max depth = 43; KNN: neighbors = 5.

**Table 2:** Performance metrics of the test set using limited dataset.

			Accuracy Mean (SD) n = 10	Precision Mean (SD) n = 10	Recall Mean (SD) n = 10	False Positive Rate Mean (SD) n = 10
Sides-Only	Baseline model	LR	0.80 (0.03)	0.79 (0.03)	0.97 (0.02)	0.60 (0.10)
		SVM	0.84 (0.06)	0.87 (0.04)	0.90 (0.06)	0.30 (0.10)
		DT	0.59 (0.08)	0.70 (0.06)	0.69 (0.10)	0.65 (0.15)
		KNN	0.62 (0.06)	0.73 (0.04)	0.71 (0.08)	0.57 (0.09)
Exclude-Diagonals	Baseline model	LR	0.78 (0.04)	0.78 (0.03)	0.94 (0.05)	0.59 (0.09)
		SVM	0.85 (0.03)	0.87 (0.02)	0.92 (0.03)	0.32 (0.06)
		DT	0.60 (0.05)	0.71 (0.04)	0.73 (0.06)	0.68 (0.12)
		KNN	0.68 (0.03)	0.69 (0.01)	0.98 (0.03)	0.98 (0.04)

Tuned hyperparameters for baseline model LR: λ = 1; baseline model SVM: C = 0.1, γ = 10; frequency domain LR: λ = 1; frequency domain SVM: C = 0.1, γ = 1 × 10^−6^; DT: max depth = 43; KNN: neighbors = 5.

## Data Availability

The data set used in the current study will be made available on request.

## Supplementary Materials

The following supporting information can be downloaded at: https://www.mdpi.com/article/10.3390/diagnostics12102404/s1, Table S1. Optimal parameters in logistic regression. Table S2. Performance metrics in the training process.
