# Supplementary material for: Detecting Parkinson’s Disease through Gait Measures Using Machine Learning
Source: Diagnostics (Basel). 2022 Oct 3;12(10):2404. doi: 10.3390/diagnostics12102404 (PMC9600300; doi:10.3390/diagnostics12102404)
Supplement: Supplementary file 1 [file diagnostics-12-02404-s001.zip › diagnostics-1847584-supplementary.pdf]

Supplementary Table S1. Optimal parameters in logistic regression.

	θ
Baseline Model	[[0.36227728 -0.27684577 0.36249594 -0.74959155 -2.36317558 -0.75735135 -1.28505679 -0.10837418 0.88861374 0.27256738 -0.28493439 -0.31584704 -0.84170895 -0.35456641 -1.20428701 -1.96159754]]
Sides Only	[[-0.09955193 0.29582405 -0.76386797 -2.6487509 -0.4904947 -1.53701646 0.17724857 0.19234567 -0.44792463 -1.04132151 -0.32247221 -1.67261833]]
Exclude Diagonals	[[0.34938102 -0.99699448 -2.58392103 -0.93272661 0.65799691 -0.35733629 -0.84651138 -2.78785022]]

Supplementary Table S2. Performance metrics in the training process.

		Accuracy Mean (SD) n=10	Precision Mean (SD) n=10	Recall Mean (SD) n=10	False Positive Rate Mean (SD) n=10
Baseline model	LR	0.82 (0.01)	0.82 (0.01)	0.95 (0.01)	0.50 (0.03)
	SVM	1.00 (0.00)	1.00 (0.00)	1.00 (0.00)	0.01 (0.01)
Frequency domain	LR	1.00 (0.002)	1.00 (0.004)	1.00 (0.003)	0.01 (0.01)
	SVM	0.99 (0.00)	0.99 (0.01)	1.00 (0.00)	0.02 (0.01)
DT		1.00 (0.002)	1.00 (0.00)	1.00 (0.003)	0.00 (0.00)
KNN		1.00 (0.002)	1.00 (0.00)	1.00 (0.003)	0.00 (0.00)
